# Case report: Successful induction of buprenorphine in medically complex patients concurrently on opioids: a case series at a tertiary care center

**DOI:** 10.3389/fphar.2024.1335345

**Published:** 2024-03-08

**Authors:** Thomas Shelton, Sharanya Nama, Orman Hall, Margaret Williams

**Affiliations:** ^1^ The Ohio State University College of Medicine, Columbus, OH, United States; ^2^ Department of Anesthesiology and Pain Medicine, The Ohio State University Wexner Medical Center, Columbus, OH, United States; ^3^ Department of Psychiatry, The Ohio State University Wexner Medical Center, Columbus, OH, United States; ^4^ Department of Internal Medicine, The Ohio State University Wexner Medical Center, Columbus, OH, United States

**Keywords:** MOUD, OUD, micro-dosing, Belbuca, buprenophine

## Abstract

Effective pain management is essential for optimal surgical outcomes; however, it can be challenging in patients with a history of opioid use disorder (OUD). Buprenorphine, a partial opioid agonist, is a valuable treatment option for patients with OUD. Initiating buprenorphine treatment in patients concurrently taking opioids can be complex due to potential adverse outcomes like precipitated withdrawal. Evolving guidelines suggest there are benefits to continuing buprenorphine for surgical patients throughout the perioperative period, however situations do arise when buprenorphine has been discontinued. Typically, in this scenario patients would be restarted on buprenorphine after they have fully recovered from post-surgical pain and no longer require opioids for pain control. Unfortunately, holding MOUD may expose the patient to risks such as opioid induced respiratory depression or addiction relapse. In this case series, we discuss a novel method to restart buprenorphine in small incremental doses, known as micro-dosing, while the patient is still taking opioids for pain. We will present two complex clinical cases when this method was used successfully at a tertiary care hospital system.

## Introduction

The opioid epidemic is one of the most pressing public health crises facing the United States today. In the 12 month period ending in December 2022, an estimated 82,310 people died from opioid overdose (CDC, 2023). This statistic marks a worrying continuation in the rise of drug overdoses seen since the onset of the COVID-19 pandemic (Centers for Disease Control and Prevention, 2020). Contributing to the increasing scale of the epidemic is the rise in prevalence of synthetic opioids such as fentanyl (CDC, 2022). The true scale of the opioid epidemic becomes clear through studies that show the true prevalence of people living with OUD is between 6–7 million (Keyes et al., 2022).

Medication treatment of Opioid Use Disorder (MOUD) has been shown to be highly effective in reducing the overall rates of mortality and relapse in patients (Sordo et al., 2017). However, there are certain circumstances when patients on MOUD are at risk for relapse. These include the induction period, the cessation of MOUD due to medical care, and the time period after hospital discharge prior to following up with outpatient addiction clinic (White et al., 2015; Sordo et al., 2017; Kohan et al., 2021). Newer research and guidelines advocate for the continuation of buprenorphine postoperatively given these risks (American Society of Addiction Medicine, 2020; Kohan et al., 2021). Additionally, it has been shown that buprenorphine may improve mortality in hospitalized patients with a history of OUD even in patients who had not been taking buprenorphine prior to hospitalization (Evans et al., 2015; Kohan et al., 2021; Button et al., 2022). Furthermore, buprenorphine treatment likely increases patient quality of life across a range of metrics (Golan et al., 2022). Unfortunately, hurdles to addiction treatment exist as many patients are hesitant to start or continue buprenorphine perioperatively due to fears of precipitated withdrawal, inadequate pain control, or limited time in the hospital (Button et al., 2022).

Historically it was recommended that buprenorphine be discontinued prior to surgery due to fears of inadequate pain control. MOUD would then be restarted once the patient no longer needed opioids for pain control. Unfortunately, perioperative discontinuation of MOUD put these patients at higher risk of OUD relapse as well as other complications such as respiratory depression (Kohan et al., 2021; Komatsu et al., 2021). To avoid these risks, micro-dosing protocols have been attempted as a way to initiate MOUD while continuing opioid medications. These protocols could allow patients to restart MOUD earlier while avoiding withdrawal symptoms, thereby reducing the risks associated with stopping buprenorphine in the perioperative period. Despite the early promise of micro induction, there is still a lack of consensus surrounding ideal dosing, time frame, and patient selection (Ahmed et al., 2021). In this case series we summarize current literature on the micro-induction of buprenorphine in the postoperative period. We also present our institution’s successful experience using patient tailored micro-induction protocols. Here, two patient cases demonstrate how micro-dosing protocols can be best applied and adapted to the individual patient.

## Current state of perioperative buprenorphine management

Buprenorphine is a highly effective drug treatment of OUD but concerns about its initiation (also known as induction) have traditionally limited its use in practice. Buprenorphine binds the μ-opioid receptor with a high affinity and thus outcompetes other opioids at the opioid receptor. When a patient has opioids in their system and has developed a tolerance to opioids, buprenorphine induction could lead to “precipitated withdrawal.” Precipitated withdrawal is the development of intense and sudden withdrawal symptoms. To avoid this, buprenorphine is typically initiated while patients are already in clinical withdrawal and have abstained from opioids (American Society of Addiction Medicine, 2020).

Though guidelines have evolved to now recommend continuation of buprenorphine in the perioperative period, variations in clinical practice still exist (Acampora et al., 2020; Wyse et al., 2022). One retrospective cohort study using Veterans Affairs Corporate Data Warehouse found that 66% of patients experienced a perioperative buprenorphine dose hold and that a year after surgery, 33% of patients lacked an active buprenorphine prescription (Wyse et al., 2022).

Patients with OUD may face more challenges with their postoperative care, like increased severity of pain following surgery and may require higher doses of opioids for adequate pain control (Cleary and Rood, 2022). In addition, nearly half of patients on MOUD may also suffer from chronic pain. (Delorme et al., 2023). Pain control perioperatively and prompt initiation of MOUD is of particular concern in this patient population. Studies have found that for patients taking MOUD, momentary pain can create cravings and may be linked to relapse (Mun et al., 2021).

Micro-induction techniques can help patients start buprenorphine perioperatively while minimizing the risks of precipitated withdrawal and effects on pain control. Such protocols are used to decrease the risk of precipitated withdrawal and allow for timely treatment of acute pain (Ahmed et al., 2021). Low doses of buprenorphine activate a few mu opioid receptors at a time minimizing symptoms of precipitated withdrawal. Furthermore, introducing small doses of buprenorphine enables clinicians to start or re-start OUD treatment earlier, therefore preventing relapse in patients whose MOUD was stopped for surgery. However, given the multifactorial nature of OUD and the lack of consensus regarding MOUD micro-induction, it is important to work with patients and tailor induction to their unique situations. This is echoed by the 2020 ASAM National Practice Guideline for the Treatment of Opioid Use Disorder which recommends that “decisions related to discontinuing or adjusting the dose of buprenorphine prior to a planned surgery should be made on an individual basis, through consultation between the surgical and anesthesia teams and the addiction treatment provider when possible” (American Society of Addiction Medicine, 2020).

## Current micro-induction strategies

The micro-induction technique is versatile and can be used to start buprenorphine for the first time, to convert MOUD from methadone to buprenophine, and in cliical scenarios with an increased risk of precipitated withdrawal. De Aquino, et al. describes success in the outpatient setting using buprenorphine transdermal patches for rapid micro-induction (De Aquino et al., 2020). Similar success was described by Silva, et al. in using the FOOT STEP protocol for outpatient micro-induction (Jasmine Silva et al., 2022). Of note, considerable variation existed among all these protocols further emphasizing the importance of adapting treatments to individual patients. Hammig, et al. describes multiple micro-dosing protocols for the re-induction of buprenorphine (Hämmig et al., 2016). Similar protocols have since been adapted in several other case studies to successfully induce and transition patients on methadone to buprenorphine (De Aquino et al., 2020; Jasmine Silva et al., 2022).

More recent studies have also explored micro-induction in the inpatient setting. DeWeese et al., reported a successful experience using an accelerated schedule in hospitalized patients. They were able to administer sizable doses over the course of 3 days and even a fully therapeutic dose of 8 mg TID over the subsequent 6 days (DeWeese et al., 2021). Another retrospective cohort study described three different micro-induction protocols. The most common method utilized in that study started buprenorphine at 0.5 mg and titrated to 4 mg BID over 6 days. A rapid-micro induction technique was also trialed which started at 0.5 mg q3-q4h. It was found that most of the patients who elected to discontinue buprenorphine initiation due to side effects were undergoing rapid-micro induction (Nunn et al., 2023). This suggests that hospital based micro-induction is feasible and effective, however careful attention to side effects is required. One characteristic that was shared by these studies was that the majority of patients on micro-induction of buprenorphine were titrated to a lower dose of 8 mg daily while on full-agonist opioids and then increased to doses between 12 and 16 mg daily once opioids were discontinued (DeWeese et al., 2021; Kohan et al., 2021).

Despite the success of micro-inductions in the literature, there remains significant variability in accepted protocol. Hjelmstrom et al.’s review of buprenorphine efficacy data could not reach any firm conclusions on dosage, or protocol, from the existing data. They recommended buprenorphine should be largely “individualized based on a continuous benefit-risk assessment” (Hjelmström et al., 2020). Together these studies show that micro-induction can be an effective tool despite the lack of consensus on protocol.

### Patient selection

Patient selection is important for successful induction of buprenorphine using micro-dosing protocol. Patients who can undergo micro-induction are those who have used illicit opioids in the preceding 5 days. Another group includes patients with OUD who are hospitalized with injury or infection causing acute pain and are receiving short acting opioids to manage pain. Using a micro-induction protocol in this circumstance decreases the 12-h waiting period after the last dose of opioid and enables quicker pain treatment. Additionally, patients who are currently on buprenorphine which has been discontinued or held for longer than 24 h over the course of treatment are also good candidates.

Micro-induction of buprenorphine is not recommended for patients who necessitate standard induction protocol. These include patients whose last illicit opioid use was more than 5 days ago and those in severe withdrawal. Patients must consent to any form of MOUD induction, and patients who have an allergy to buprenorphine buccal film or patch are also not candidates for micro-induction.

### Variable dosing schedules

**FIGURE 1:**
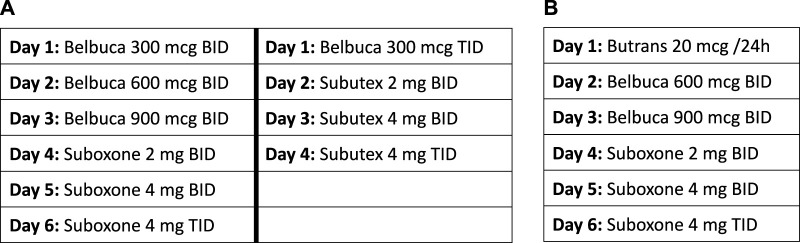
The Ohio State University variable dosing schedules. **(A)** Example dosing schedule utilizing buccal film. **(B)** Example dosing schedule utilizing buccal film and transdermal patch.

The Ohio State University Medical Center (OSUMC) addiction service has utilized various micro-dosing schedules. Patient can be initially started on buprenorphine buccal film (Belbuca^™^) and then transitioned to sublingual buprenorphine formulations (Suboxone^™^, Subutex^™^). Alternatively, patients can be started on a buprenorphine transdermal patch (Butrans^™^), then transitioned to buprenorphine buccal and then ultimately switched to sublingual buprenorphine formulations. Examples of the most commonly used dosing schedules are outlined in Figure 1.

## Case series

### Case 1

#### Patient information and clinical findings

**FIGURE 2:**
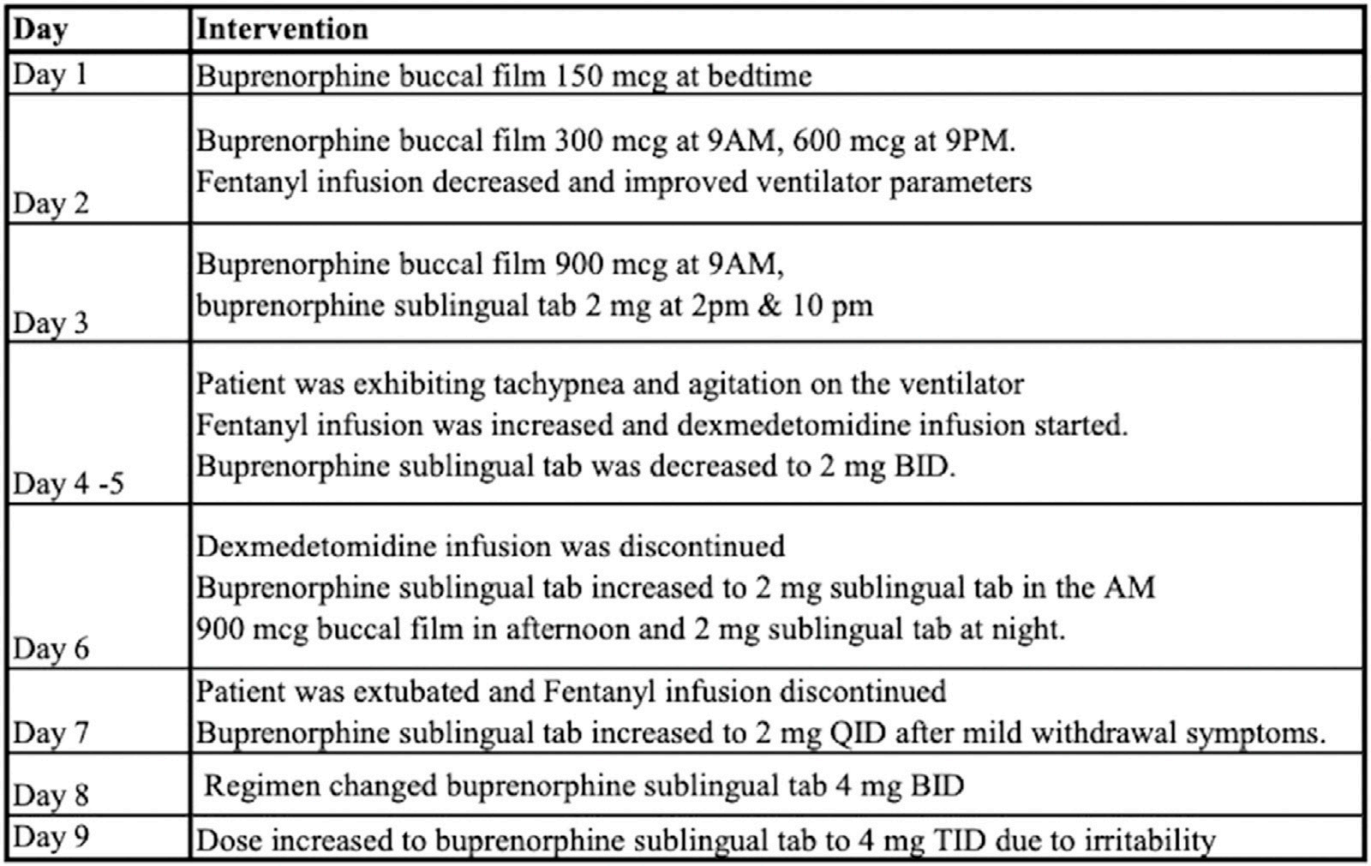
Timeline and Therapeutic Intervention—Micro-dosing regimen Case 1.

Patient 1 is a critically ill female in her thirties transferred to our institution from an outside facility for management of sepsis and respiratory failure due to pneumonia. Upon arrival she was mechanically ventilated and in septic shock requiring vasopressor support and broad-spectrum antibiotics. Pertinent medical history includes OUD treated with maintenance buprenorphine-naloxone which was discontinued. She was sedated with fentanyl and midazolam infusions. Later in the hospitalization, the addiction service was consulted for recommendations on restarting MOUD. Review of her prescription drug monitoring program history revealed that she had consistently been on buprenorphine-naloxone 8–2 mg daily. After discussion with immediate family, it was determined that restarting MOUD was in line with the patient’s treatment goals. A micro-dosing protocol was tailored for this specific clinical circumstances and is outlined in Figure 2. While the patient was on the micro-dosing protocol, breakthrough pain was controlled with short acting full agonist opioids (ex. hydromorphone) and medications for the treatment of opioid withdrawal were administered when needed.

#### Outcomes and follow-up

Patient was discharged home on HD17 on buprenorphine sublingual tab 4 mg TID with outpatient follow up schedule with addiction clinic.

### Case 2

#### Patient information and clinical findings

Patient 2 is a male in his late thirties, with unknown past medical history upon admission to an outside hospital sustaining injuries after a motor vehicle accident. The patient presented obtunded with a Glasgow Coma Scale 5 and was intubated. Workup revealed facial, ankle and rib fractures, a left sided pneumothorax, and a subarachnoid hemorrhage. In addition, he sustained injury to the right iliac artery and was transferred to our institution for further management.

He underwent multiple procedures including:

HD1: IR embolization of iliac artery. External fixation of left ankle and closed reduction of right hip with traction pinning.

HD6: Tracheostomy, ORIF of Le Fort facial fractures, dental extractions, and PEG tube placement.

HD7: Hip acetabular fracture repair.

HD15: Exploratory laparotomy and splenectomy after development of acute abdomen and hemorrhagic shock.

HD16: Abdominal wound closure.

HD18: ORIF ankle fracture.

**FIGURE 3:**
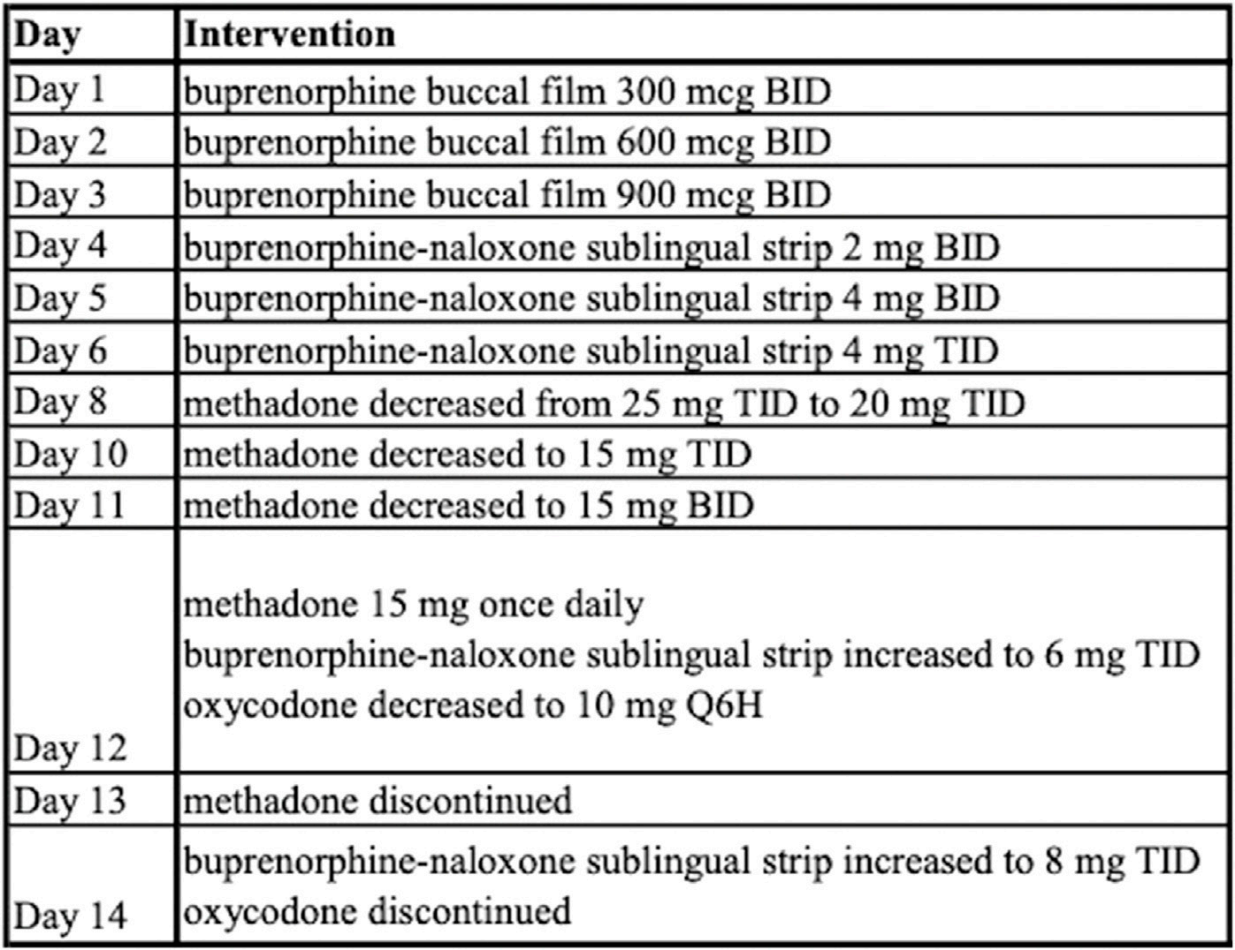
Timeline and Therapeutic Intervention—Micro-dosing regimen.

The addiction service was consulted on day 24 and the patient discussed history of substance use disorder and consented to buprenorphine initiation. A buprenorphine micro-dosing schedule was initiated as outlined in Figure 3. The patient’s pain was managed initially with hydromorphone and ketamine infusions along with oral methadone 25 mg TID. He was then transitioned to intermittent doses of intravenous hydromorphone and scheduled oral oxycodone which was tapered and discontinued prior to discharge.

#### Outcomes and follow-up

Patient discharged home on day 43 with prescription for buprenorphine-naloxone sublingual strip 8 mg TID with follow up planned at a local addiction clinic.

## Discussion/conclusion

The current literature supports continuing buprenorphine during episodes of acute pain as it offers distinct advantages (Kohan et al., 2021). By capitalizing on buprenorphine’s unique pharmacological properties, clinicians can achieve effective pain control while minimizing the risk of opioid withdrawal, and potentially use buprenorphine as a bridge between analgesia and addiction management (Ahmed et al., 2021; Edinoff et al., 2023). This approach aligns with the overarching shift towards individualized pain management paradigms and reinforces the importance of personalized care plans.

Specific to micro-induction, the existing body of literature is conflicted on its utility. A recent systematic review of buprenorphine micro-induction by Spreen et al., concluded that micro-induction protocols were comparable to traditional initiation protocols, and effectively reduce withdrawal symptoms (Spreen et al., 2022). However, a separate review and pharmacological model suggests that micro-induction protocols may have limited use, and that traditional induction may be a more effective method of induction in many settings (Greenwald et al., 2022). Given the ongoing controversy in the field, case series like this one offer additional value in contributing to this rapidly evolving literature.

Our case series provides a tangible example of the successful micro-dosing of buprenorphine in patients on concurrent full opioid agonists. The seamless integration of buprenorphine within a multimodal analgesic regimen resulted in optimal pain relief, mitigated opioid cravings, and facilitated a smoother recovery process. However, the case also highlights the significance of meticulous patient selection, interdisciplinary collaboration, and judicious dose adjustment to ensure patient safety and favorable outcomes. This is consistent with literature reviews, which emphasize the importance of protocol flexibility to treat patients most effectively across wide ranges of settings (Ahmed et al., 2021).

While the reviewed evidence and case studies underscore the potential benefits, it is crucial to acknowledge the complexities associated with continuing buprenorphine in the setting of acute pain. Challenges such as individual variability in response, potential drug interactions, and the need for clear communication among clinicians is paramount (Spreen et al., 2022; Edinoff et al., 2023). Further research is warranted to delve into the nuances of dosing strategies, patient selection criteria, and the long-term impact of this approach on pain trajectories and addiction management. As the medical community continues to advance in its understanding addiction treatment, collaborative efforts will play a pivotal role in shaping its integration strategies like micro-induction, thereby optimizing outcomes for patients facing the intersection of acute pain and opioid use disorder.

## Data Availability

The original contributions presented in the study are included in the article/supplementary material, further inquiries can be directed to the corresponding author.
